# Pharmacokinetics of meropenem in septic patients on sustained low-efficiency dialysis: a population pharmacokinetic study

**DOI:** 10.1186/s13054-018-1940-1

**Published:** 2018-01-30

**Authors:** Stephan Braune, Christina König, Jason A. Roberts, Axel Nierhaus, Oliver Steinmetz, Michael Baehr, Stefan Kluge, Claudia Langebrake

**Affiliations:** 1 Department of Intensive Care Medicine, University Medical Center Hamburg-Eppendorf, Hamburg, Germany; 2 Hospital Pharmacy, University Medical Center Hamburg-Eppendorf, Hamburg, Germany; 3 University of Queensland Centre for Clinical Research, Faculty of Medicine and Centre for Translational Anti-infective Pharmacodynamics, School of Pharmacy, The University of Queensland, Brisbane, Australia and Royal Brisbane and Women’s Hospital, The University of Queensland, Brisbane, QLD Australia; 4 III. Medical Clinic and Polyclinic, Department of Nephrology, University Medical Center Hamburg-Eppendorf, Hamburg, Germany; 5 Department of Stem Cell Transplantation, University Medical Center Hamburg-Eppendorf, Hamburg, Germany

**Keywords:** Pharmacokinetics, Meropenem, Sepsis, Acute renal failure, Sustained low-efficiency dialysis

## Abstract

**Background:**

The aim of the study was to describe the population pharmacokinetics (PK) of meropenem in critically ill patients receiving sustained low-efficiency dialysis (SLED).

**Methods:**

Prospective population PK study on 19 septic patients treated with meropenem and receiving SLED for acute kidney injury. Serial blood samples for determination of meropenem concentrations were taken before, during and after SLED in up to three sessions per patient. Nonparametric population PK analysis with Monte Carlo simulations were used. Pharmacodynamic (PD) targets of 40% and 100% time above the minimal inhibitory concentration (*f* T _> MIC_) were used for probability of target attainment (PTA) and fractional target attainment (FTA) against *Pseudomonas aeruginosa*.

**Results:**

A two-compartment linear population PK model was most appropriate with residual diuresis supported as significant covariate affecting meropenem clearance. In patients without residual diuresis the PTA for both targets (40% and 100% *f* T _> MIC_) and susceptible *P. aeruginosa* (MIC ≤ 2 mg/L) was > 95% for a dose of 0.5 g 8-hourly. In patients with a residual diuresis of 300 mL/d 1 g 12-hourly and 2 g 8-hourly would be required to achieve a PTA of > 95% and 93% for targets of 40% *f* T _> MIC_ and 100% *f* T _> MIC_, respectively. A dose of 2 g 8-hourly would be able to achieve a FTA of 97% for 100% *f* T _> MIC_ in patients with residual diuresis.

**Conclusions:**

We found a relevant PK variability for meropenem in patients on SLED, which was significantly influenced by the degree of residual diuresis. As a result dosing recommendations for meropenem in patients on SLED to achieve adequate PD targets greatly vary. Therapeutic drug monitoring may help to further optimise individual dosing.

**Trial registration:**

Clincialtrials.gov, NCT02287493.

## Background

In the intensive care unit (ICU), up to 42% of septic patients develop acute kidney injury (AKI) with approximately 5% of all ICU patients requiring renal replacement therapy (RRT) [1, 2]. Because of their high mortality risk optimised antibiotic dosing in these patients is considered mandatory to improve clinical outcomes [3, 4].

Meropenem is a broad-spectrum antibiotic agent commonly used in critically ill patients [5–7]. It has minimal protein binding (2%) and is mainly excreted unchanged in the urine (approximately 70% unchanged; 28% as inactive metabolite) [5, 7]. In patients with normal kidney function, meropenem has a half-life of approximately 1 h increasing to > 5.7 h with AKI [5]. Meropenem has a time-dependent bacterial killing characteristic, which means a plasma concentration above the minimum inhibitory concentration (MIC) for at least 40% of time of the dosing interval (40% *f* T _> MIC_) is associated with optimal activity [6–9]. Recent studies suggest a target of 100% *f* T _> MIC_ to be more appropriate for critically ill patients with severe sepsis [10–12].

In addition to the alterations of meropenem pharmacokinetics (PK) caused by sepsis, use of RRT has additional profound effects on drug clearance (CL) [13–22]. These effects on PK differ according to the modality and intensity of RRT [21, 23–27].

Sustained low-efficiency dialysis (SLED) is an increasingly used modality of prolonged intermittent RRT in the ICU [28–30]. SLED combines both the advantage of intermittent haemodialysis (IHD) with its good solute removal and that of continuous renal replacement therapy (CRRT) with its haemodynamic stability [31–33]. Because of its intermittent application, the PK of drugs with predominantly renal elimination change from high CL during SLED to a significantly lower CL without SLED.

Despite its widespread use, the lack of SLED-specific dosing studies leave intensivists with considerable uncertainty about optimal antibiotic dosing [14, 25, 29, 30, 34–37]. So far, only two small studies on meropenem PK during SLED have been published and neither of them described PK over serial SLED treatments nor used a population PK approach to propose SLED-specific meropenem dosing regimens [38, 39]. The aim of this study was to describe the population PK of meropenem in critically ill septic patients receiving SLED and to develop dosing recommendations for meropenem in this population.

## Methods

### Setting

This was a prospective, observational population PK study. Patients were recruited between July 2013 and November 2014 in the Department of Intensive Care Medicine at the University Medical Center Hamburg-Eppendorf in Germany. Ethics approval was obtained from the local ethics committee. Written informed consent was obtained either from the patient or their appointed legal guardian. The study was registered at clincialtrials.gov (NCT02287493).

### Study population

Patients aged > 18 years treated with meropenem and receiving SLED during daytime were eligible for study inclusion.

### Dosing, administration and data collection

Dosing was at the discretion of the treating physician with various doses of 0.5 g, 1 g or 2 g of meropenem (Dr. Friedrich Eberth Arzneimittel GmbH, Germany) administered intravenously over 30 minutes 8-hourly.

Demographic data and clinical variables such as residual diuresis, mechanical ventilation, vasopressor support, modified sequential organ failure assessment (SOFA) score on the first day of sampling, simplified acute physiology (SAPS II) score on ICU admission, as well as laboratory variables, such as liver enzymes, C-reactive protein (CRP) and procalcitonin (PCT), were collected. Patient outcome data including ICU length of stay as well as ICU mortality were documented.

### Sustained low-efficiency dialysis

SLED was performed with the Genius® batch system (Fresenius Medical Care, Bad Homburg, Germany) using a Fresenius FX 60 filter (surface area 1.4 m^2^, Fresenius Medical Care). Blood/dialysate and ultrafiltration flow rates as well as the duration of SLED were recorded.

### Sample collection and measurements

Blood sampling from an indwelling cannula was performed on three consecutive days of SLED. Trough samples 1 h prior to infusion were taken followed by sampling 10 min and 1 h, 2 h and 4 h after the start of SLED, as well as at the end of the session. SLED was initiated no later than 3 h after meropenem infusion. Blood samples were centrifuged within 30 minutes at 3000 rpm, for 10 minutes. The serum supernatant was transferred to a tube and stored at –70 °C until analysis. According to previous stability testing, we set a maximum storage time of 3 months [40]. Samples were analysed by high-performance liquid chromatography with UV-detection (HPLC-UV) after being processed for protein precipitation. The method was validated and conducted in accordance with the guidelines of the US Food and Drug Administration’s guidance for industry on bioanalysis [41]. The coefficient of variation for the intra-day precision was 9.6%, 3.9%, and 2.2% for meropenem concentrations of 10, 20, and 80 mg/L. Inter-day precision coefficients of variation were < 15% for 10, 20, and 80 mg/L. Accuracy was > 94% with a deviation of < 15% for all tested concentrations including the lower limit of quantification of 1 mg/L.

### Population pharmacokinetic modeling

One and two-compartment models were tested using the Nonparametric Adaptive Grid (NPAG) algorithm within the Pmetrics® package for R (Los Angeles, CA, USA) [42, 43]. Additive (lambda) and exponential (gamma) error models were both tested for inclusion. Demographic and clinical characteristics, which were considered biologically plausible for affecting meropenem PK, such as residual diuresis, blood/dialysate flow and bodyweight, were tested for inclusion as covariates. If a covariate improved the coefficient of determination of the linear regression (R^2^) and resulted in the reduction of the bias of the goodness-of-fit plots as well as in a statistically significant reduction in the log-likelihood (*p* < 0.05) it was included in the model. The R^2^ and the bias of the observed versus predicted plots as well as the log-likelihood of each run were taken into account for the goodness-of-fit evaluation. Predictive performance evaluation was based on mean prediction error (bias) and the mean bias-adjusted squared prediction error (imprecision) of the population and individual prediction models. Weighted residual plots versus time and concentration, as well as the visual predictive check (VPC) plot and the normalised prediction distribution errors (NPDE) were also used to test the suitability of the final covariate model.

### Probability of target attainment

Monte Carlo simulations (*n* = 1000) were performed using Pmetrics® to determine the probability of target attainment (PTA) for the first 24 h of meropenem treatment for two pharmacodynamic (PD) targets. The primary PD target was set to the traditional target of 40% *f* T _> MIC_ [8]. Additionally, a more aggressive and higher target was set to 100% *f* T _> MIC_ [10, 11]. We simulated a 5-hour SLED treatment between the administration of 0.5 to 2 g meropenem 8-hourly and 1 to 2 g 12-hourly with an infusion time of 30 minutes. In addition we simulated both prolonged (3 hours) and continuous infusion (CI) of meropenem. In the simulation, SLED started 17 h after the first dose of meropenem, translating to a SLED session between the 2^nd^ and the 3^rd^ dose for 8-hourly dosing and at the end of the dosage interval for a 12-hourly dosing interval. The PTA against various MIC accounted for the plasma protein binding of 2% [5–7]. A dosage regimen was considered successful with a PTA > 95%.

### Fractional target attainment

The fractional target attainment (FTA) identifies the achievement of target antibiotic exposures by comparing the pharmacodynamic exposure (PTA) against an MIC distribution of a chosen bacteria. To determine the FTA against *P. aeruginosa* with a susceptibility breakpoint of 2 mg/L, MIC data from the EUCAST database were used [8]. Additionally, MIC ≤ 8 mg/L and MIC ≤ 16 mg/L were also tested to account for P. *aeruginosa* strains at the resistance breakpoint. The FTA was calculated by using the targets of 40% *f* T _>MIC_ and 100% *f* T _>MIC_. A dosing regimen was considered successful if the FTA was > 95%.

## Results

### Demographic and clinical data

A total of 308 serum samples were obtained from 19 patients. Seventy-four percent were male patients with a median [range] age, weight and SOFA score of 66 years [37–78], 81 kg [70–183] and 11 [5–16], respectively. The median residual diuresis and SLED treatment time were 0 mL/day [0–360] and 315 min [80–470], respectively. Detailed demographic and clinical data are shown in Table 1.

**Table 1:** Demographic and clinical data

Variable	Mean (SD), n (%) or median [IQR]	Range
Age, years	66 [51–74]	37–78
Male gender	14 (74%)	N/A
Height, m	1.73 [1.68–1.82]	1.55–1.90
Weight, kg	81 [76–90]	70–183
BMI, kg/m^2^	28 [26–31]	22–57
SOFA score on ICU admission	10 [9–13]	2–20
SAPS II on ICU admission	46 [34–50]	16–76
SOFA score on the first day of sampling	11 [9–13]	5–16
Mechanical Ventilation on sampling day 1	27 (58%)	N/A
Use of Vasopressors on sampling day 1	17 (89%)	N/A
Procalcitonin [μg/L] on sampling day 1	1.48 [0.72–3.02]	0.28–21.4
C-reactive protein on sampling day 1	101 [51–162]	16–267
Serum creatinine [mg/dL]* on sampling day 1	2.6 [1.7–4]	0.8–9.1
Serum albumin conc. [g/L] on sampling day 1	15.5 (3.7)	10–21
Meropenem trough concentration [mg/L]	28.9 [21.6–36.9]	10.2–95.8
SLED duration [min]	315 [275–354]	80–470
Residual diuresis [mL/d]	0 [0-80]	0–360
Blood/dialysate flow [mL/min]	250 [208–278]	170–350
Ultrafiltration rate [mL/h]	500 [400–597]	50–1000
ICU length of stay [days]	36 [23–79]	8–264
Death	9 (47%)	N/A

*BMI* body mass index, *ICU* intensive care unit, *IQR* interquartile range, *N/A* not applicable, *SD* standard deviation, SLED sustained low-efficiency dialysis, SAPS II Simplified Acute Physiology Score; SOFA sequential organ failure assessment, *Data of the first day of inclusion in study, possibly affected by previous SLED or CRRT sessions

### Pharmacokinetic model building

A two-compartment linear model using an additive error adequately described the serum concentrations of meropenem. Residual diuresis was found to be associated with CL when SLED was not being used and improved the goodness-of-fit and the log-likelihood (*p* < 0.01) of the model. No other covariates could be identified as significant, e.g. blood/dialysate and ultrafiltration flow rate. The final model is described as follows:

$$ {\displaystyle \begin{array}{c} TVC L=C{{\mathrm{L}}_{\mathrm{SLED}}}^{\ast}\left(\mathrm{SLED}\right)+C{\mathrm{L}}_{\mathrm{N}\mathrm{S}}\\ {} TVC{\mathrm{L}}_{\mathrm{N}\mathrm{S}}=C{\mathrm{L}}_{\mathrm{D}}+\left({{\mathrm{CL}}_{\mathrm{N}}}^{\ast}\mathrm{RD}/100\right)\end{array}} $$

Where TVCL is the typical value of total CL, CL_SLED_ is the population parameter estimate of meropenem CL with SLED. CL_NS_ is the population parameter estimate of meropenem CL without SLED. The value for term SLED is 1 when SLED is on, whereas it is 0 when SLED is off. CL_D_ is the meropenem CL referring to non-SLED CL mechanisms. CL_N_ is the native meropenem CL associated with the residual diuresis (RD) of the patient.

The mean (SD) population PK parameter estimates from the final covariate model are displayed in Table 2.

**Table 2:** Parameter estimates for meropenem from the final covariate two-compartment population pharmacokinetic model

Variable	Mean	Standard deviation	Coefficient of variation (%)	Median
CL_SLED_ (L/h)	7.9	4.2	53.6	6.8
CL_N_ (L/h)	1.5	2.1	134.7	0.7
CL_D_ (L/h)	2.6	1.2	44.9	2.3
V_c_ (L)	8.1	7.1	87.9	4.9
KCP	10.3	8.8	85.4	7.9
KPC	1.8	1.9	104.4	1.2

CL_SLED_ clearance on SLED, CL_N_ clearance native, CL_D_ clearance related to other mechanisms, V_c_ volume of distribution of the central compartment, KCP constant for the distribution of meropenem from the central to the peripheral compartment, KPC constant for the distribution of meropenem from the peripheral to the central compartment

**Fig. 1:**
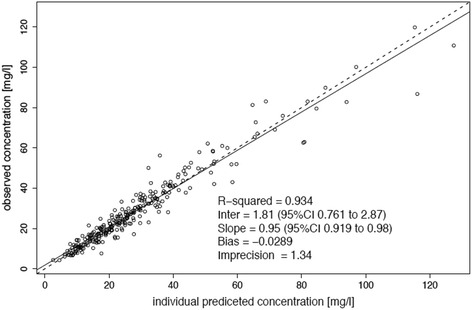
Diagnostic plots for the final covariate model. Observed versus individual predicted concentrations

**Fig. 2:**
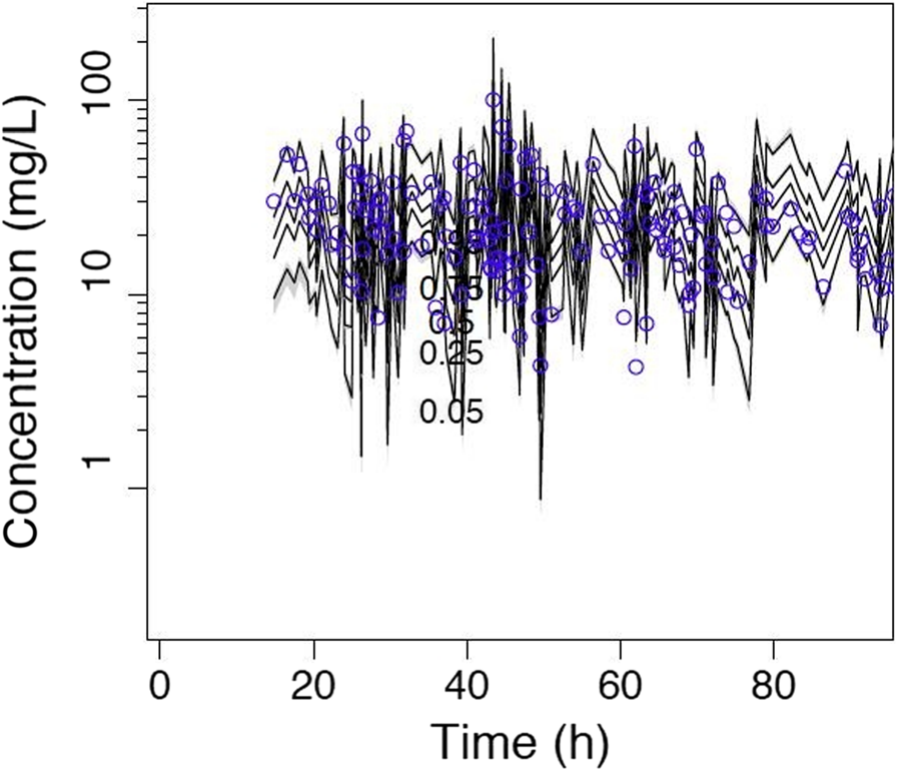
Diagnostic plots for the final covariate model. Visual predictive checks

The diagnostic plots confirmed the goodness-of-fit of the chosen model and are shown in Figs. 1 and 2. Monte Carlo simulations were performed with the final covariate model.

### Probability of target attainment

**Fig. 3:**
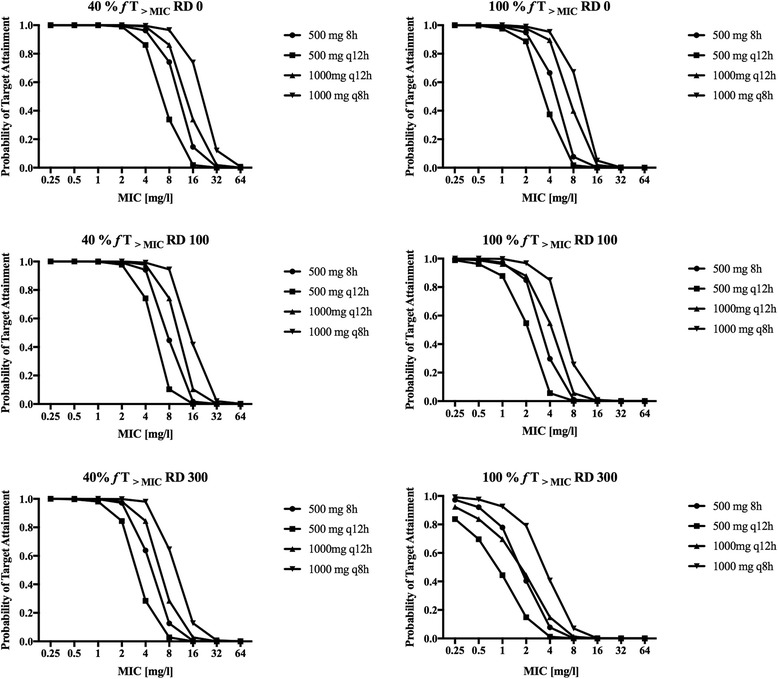
Probability of target attainment (PTA) of various meropenem doses for a residual diuresis (RD) of 0 mL/d, 100 mL/d and 300 mL/d for a PD target of 40% *f* T _> MIC_ (*left column*) and for a PD target of 100% *f* T _> MIC_. (*right column*)

The probability of target attainment (PTA) for achieving 40% and 100% *f* T _>MIC_ for the first 24 h for various meropenem doses in patients receiving a 5-hour SLED session are described in Fig. 3. These plots generally show that the PTA decreases with a higher residual diuresis. Regarding 40% *f* T _> MIC_ with various residual diuresis up to 300 mL/d 0.5 g 8-hourly achieved a PTA of > 95% for MIC ≤ 2 mg/L (Fig. 3, left column).

**Fig. 4:**
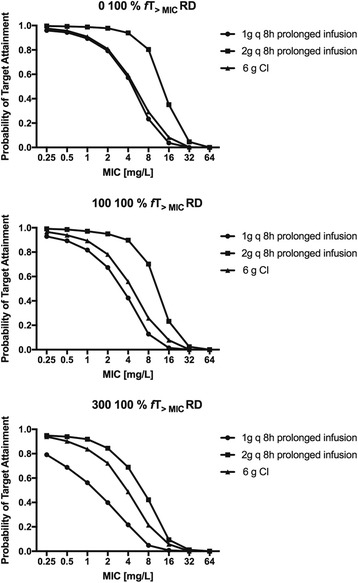
Probability of target attainment (PTA) prolonged (1 g and 2 g 8-hourly) and continuous meropenem (6 g/24 hours) infusions for a residual diuresis (RD) of 0 mL/d, 100 mL/d and 300 mL/d for a PD target of 100% *f* T _> MIC_

**Fig. 5:**
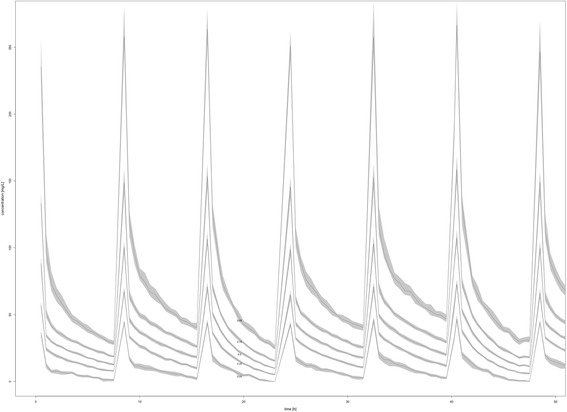
PK profile of meropenem at a dose of 2 g 8-hourly in patients with a residual diuresis of 300 ml/d. Time (hours) displayed on the x-axis, concentrations (mg/L) displayed on the y-axis

However, aiming for 100% *f* T _> MIC_ with a residual diuresis of 0 mL/d a dose of 1 g 12-hourly resulted in a sufficient PTA above 95%. With a residual diuresis of 300 mL/d a dose of 2 g 8-hourly only resulted in a PTA of 93% (Fig. 3, right column). The results for modelling prolonged (3 hours) and continuous meropenem infusions for a PK target of 100% *f* T _> MIC_ are shown in Fig. 4. The PK profile of meropenem at a dose of 2 g 8-hourly in patients with a residual diuresis of 300 ml/d is shown in Fig. 5.

### Fractional target attainment

The FTAs for 40% and 100% *f* T _> MIC_ for the first 24 h and a 5-hour SLED session for a range of meropenem doses and degrees of residual diuresis for susceptible *P. aeruginosa* isolates (MIC ≤ 2 mg/L) are shown in Table 3. The FTA against *P. aeruginosa* strains at and below the resistance breakpoint (MIC ≤ 8 mg/L) and for strains with a MIC of ≤ 16 mg/L are shown in Tables 4 and 5, respectively. In the simulations for these two MICs we included analyses of prolonged infusions (3 h) of meropenem with the highest dose of 2 g 8-hourly.

**Table 3:** Fractional target attainment (FTA) for directed therapy for susceptible *P. aeruginosa* (MIC < 2 mg/L, 45,715 isolates)

RD [mL/d]	Dose (40% *f* T _> MIC_)					Dose (100% *f* T _> MIC_)				
	0.5 g q12	0.5 g q8	1 g q12	1 g q8	2 g q8	0.5 g q12	0.5 g q8	1 g q12	1 g q8	2 g q8
0	99.9	99.9	100	100	100	94.0	99.3	99.7	99.9	100
100	99.7	99.9	99.9	100	100	91.3	97.5	97.5	99.6	99.9
300	97.8	99.6	99.7	99.9	100	67.8	86.3	81.8	86.6	97.0

RD residual diuresis, *f* T _> MIC_ percentage of time remaining concentration above MIC

**Table 4:** Fractional target attainment (FTA) for empiric therapy for *P. aeruginosa* strains at and below the resistance breakpoint (MIC ≤ 8 mg/L, 52,771 isolates)

RD [mL/d]	Dose (40% *f* T _> MIC_)						Dose (100% *f* T _> MIC_)					
	0.5 g q12	0.5 g q8	1 g q12	1 g q8	2 g q8	2 g Q8 PI	0.5 g q12	0.5 g q8	1 g q12	1 g q8	2 g q8	2 g Q8 PI
0	94.8	98.1	99.1	99.8	99.9	100	87.9	91.2	95.3	97.5	99.6	97.7
100	92.4	96.1	98.2	99.6	99.9	99.9	79.5	86.7	88.8	93.9	98.8	95.9
300	86.9	91.7	94.2	97.7	99.9	99.9	58.8	75.3	71.9	75.5	92.2	94.3

RD residual diuresis, *f* T _> MIC_ percentage of time remaining concentration above MIC, PI prolonged infusion (3 h)

**Table 5:** Fractional target attainment (FTA) for empiric therapy for *P. aeruginosa* strains over the resistance breakpoint (MIC < 16 mg/L, 56,730 isolates)

RD [mL/d]	Dose (40% *f* T _> MIC_)						Dose (100% *f* T _> MIC_)					
	0.5 g q12	0.5 g q8	1 g q12	1 g q8	2 g q8	2 g q8 PI	0.5 g q12	0.5 g q8	1 g q12	1 g q8	2 g q8	2 g q8 PI
0	88.3	92.3	94.5	97.9	99.7	99.9	81.8	84.9	88.8	91.1	97.4	93.3
100	85.9	89.5	92.1	95.6	99.56	99.8	73.9	80.7	82.6	87.5	93.7	90.9
300	80.0	85.3	87.8	91.7	95.9	99.4	54.7	70.0	66.9	70.3	86.3	83.4

RD residual diuresis, *f* T _> MIC_ percentage of time remaining concentration above MIC, PI prolonged infusion (3 h)

For susceptible strains (45,715 isolates), 0.5 g 8-hourly achieved an average FTA of > 95% both for the traditional target of 40% *f* T _> MIC_ and the aggressive target of 100% *f* T _> MIC_ in patients without residual diuresis (Table 3). A residual diuresis of 100 or 300 mL/d did not change the results for the traditional target of 40% *f* T _> MIC_. In contrast, in simulations with a residual diuresis of 300 mL/d and with setting the target to 100% *f* T _> MIC_ a meropenem dose of 2 g 8-hourly achieved an average FTA of 97%.

In the context of *empiric* therapy which includes *P. aeruginosa* strains at and below the resistance breakpoint (MIC ≤ 8 mg/L, 52771 isolates) a dose of 0.5 g 8-hourly achieved a FTA of > 95% for 40% *f* T _> MIC_ in patients with residual diuresis of 0 and 100 mL/d, respectively (Table 4). For the more aggressive target of 100% *f* T _> MIC_, a dosing regimen of 2 g 8-hourly resulted in a FTA of 99% in patients with a residual diuresis ≤ 100 mL/d. In patients with a residual diuresis of 300 mL/d a meropenem dose of 2 g 8-hourly achieved a FTA of 92% (Table 4). This was further improved to 95% when simulating for prolonged infusions. In more resistant strains (MIC ≤ 16 mg/L, 56730 isolates) aiming at a PD target of 40% *f* T _> MIC_ a meropenem dose of 2 g 8-hourly achieved a FTA of 95% with a residual diuresis of 300 mL/d. When aiming for a PD target of 100% *f* T _> MIC_, the highest meropenem dose of 2 g 8-hourly only resulted in a FTA > 95% in patients without residual diuresis (Table 5).

## Discussion

This is the first study to describe the PK of meropenem in *serial* SLED treatments of critically ill septic patients in AKI using a population PK approach. The results show that achievement of optimal meropenem dosing in septic patients treated with SLED, depends on the microbiological susceptibility, the PK/PD-target (40% vs. 100% *f* T _> MIC_) and on the degree of residual diuresis.

Meropenem has been shown to have a CL that varies with the mode of RRT [24]. Therefore PK data from studies that did not include patients receiving SLED cannot be used to guide dosing during SLED. Under continuous venovenous haemofiltration (CVVH) and continuous venovenous haemodiafiltration (CVVHDF), mean meropenem CL was described to be 1.9 L/h and 3.6 L/h, respectively [44–46]. The meropenem CL on SLED (CL_SLED_ = 7.9 L/h; ± 4.2 L/h) in this study was substantially higher than those reported for continuous RRT [44–47]. This can be explained by higher effluent and blood flow rates on SLED. In fact, CL_SLED_ for meropenem was higher than the CL under intermittent RRT reported from Christensson et al. and Chimata et al. (1.2 and 4.8 L/h) who used cuprophan haemodialysis filters, which are not comparable to our filter systems [48, 49]. Compared to healthy volunteers (11–14 L/h), meropenem CL is significantly lower during SLED (7.9 L/h) [48, 49].

Not surprisingly, being on or off SLED had most impact on attaining target meropenem exposures in our model. The model fit further significantly increased by including the native clearance (CL_N_) linked to the residual diuresis. This is plausible since residual diuresis leads to additional meropenem elimination. This relationship has previously been shown by Ulldemolins et al. [50].

Due to the design of the PK model, CL without SLED (CL_NS_) is composed of CL_N_ (1.5 L/h) and CL_D_ (2.6 L/h) and is higher than Christenssen et al. reported within patients with end stage renal disease (CL = 1.2 L/h) [48]. This may be related to the fact, that SLED in our clinical setting was sometimes used to maintain fluid balance rather than for solute clearance. Additionally, carrying over effects of previous CRRT sessions as well as non-steady state conditions in intervals without SLED could have influenced these CL. The central volume of distribution (V_c_) in our patients of 8.1 L (±7.1) was in accordance with the data of Roberts et al. who found a V_c_ of 7.9 L in septic patients [51].

A dosage regimen of 0.5 g 8-hourly would be adequate to achieve a PTA > 95% for the conventional target of 40% *f* T _> MIC_ irrespective of the residual diuresis. However, for a target of 100% *f* T _> MIC_ and a residual diuresis of up to 300 mL/d a dose of 2 g 8-hourly, which is the approved upper dosage limit, would achieve a PTA of 93%. Our results also showed that neither prolonged nor continuous meropenem infusion increased PTA compared with bolus application. This can be explained by the fact that in patients with AKI treated with SLED the meropenem clearance over a 24-hour period mainly occurs during SLED.

Regarding the FTA while aiming for the traditional target of 40% *f* T _> MIC_ for susceptible *P. aeruginosa* (MIC ≤ 2 mg/L) a reduced dose of 0.5 g 8-hourly would ensure sufficient bactericidal activity irrespective of residual diuresis. As opposed to this, for a more aggressive target of 100% *f* T _> MIC_ for susceptible *P. aeruginosa* (MIC ≤ 2 mg/L) an increased dose of 1 g 8-hourly would be required for patients with a residual diuresis of ≤ 100 mL/d. Further differing from this, patients with a residual diuresis of 300 mL/d would require the maximally approved dose of 2 g 8-hourly to achieve the target of 100% *f* T _> MIC_ (FTA 97%). However, for *P. aeruginosa* strains at and below the resistance breakpoint (MIC ≤ 8 mg/L) we found that in patients with a residual diuresis of up to 300 mL/d even the maximally approved dosage of 2 g 8-hourly would result in an FTA of only 92%. The FTA was increased to 95% in this setting by simulating prolonged infusion (3 h) of 2 g 8-hourly.

Finally, for *P. aeruginosa* strains with a MIC ≤ 16 mg/L the maximally approved dose of 2 g 8-hourly only led to a target FTA (>95%) when aiming at a PK target of 40% *f*T _> MIC_. This was further improved by prolonged infusions in patients with a residual diuresis of 300 ml/d. When aiming for a target of 100% *f*T _> MIC_ the maximal dose of 2 g 8-hourly only achieved a FTA > 95% in patients without residual diuresis. Prolonged infusions had no effect in this setting. When applying high doses of meropenem to ensure bactericidal activity as suggested by our simulations, clinicians need to balance this benefit against potential side effects such as seizures. However, the incidence of meropenem-associated seizures is described as low (<1%) and they are usually reversible on discontinuation and manageable with anticonvulsants [52].

### Limitations

Endogenous renal function could not be estimated using standard approaches because serum creatinine as well as meropenem concentrations were affected by previous SLED or CRRT sessions.

Additionally, post-SLED treatment samples were not collected. Therefore, a potential rebound in serum concentrations as it has been described for vancomycin could have been missed, although this effect has not be shown for substances such as meropenem [39]. Therefore, this effect is not likely to affect the recommended dosing regimens.

Our study only simulates for 5 hours of SLED, as this was the average duration of SLED in our department, limiting extrapolation to patients receiving longer and more intensive SLED. The relatively short duration of SLED with the Genius® device in our routine clinical setting was often due to unintended interruptions for various reasons such as malfunctions of the extracorporeal circuit or early termination for patient mobilisation or transport. However, this often reflects common practice with SLED in the ICU.

Furthermore, simulating extended durations of SLED with our model would have increased the overall meropenem clearance per SLED session and thus may potentially underestimate dosing recommendations. Finally, some patients were treated with a CRRT before switching to SLED, which potentially could have led to carrying over effects from the previous CRRT.

## Conclusions

In this study, we observed a relevant PK variability for meropenem in patients on SLED, which was significantly influenced by the degree of residual diuresis. As a result the given dosing recommendations for meropenem in patients on SLED to achieve adequate PD targets greatly vary. Patients with high residual diuresis, proven or suspected pathogens strains at and below the resistance breakpoint as well as aggressive PD targets require the maximum approved dosages. Further studies are required to validate our findings.

Given the high variability of meropenem PK under RRT and bacterial susceptibility, therapeutic drug monitoring may help to further optimise individual meropenem dosing in clinical practice [4, 25, 53].

## Abbreviations

AKI: Acute kidney injury
BMI: Body mass index
CI: Continuous infusion
CL: Clearance
CL_SLED_: Meropenem clearance on SLED
CL_NS_: Population parameter estimate of meropenem clearance without SLED
CL_D_: Meropenem clearance referring to non-SLED clearance mechanisms
CL_N_: Native meropenem clearance associated with the residual diuresis
CRP: C-reactive protein
CRRT: Continuous renal replacement therapy
CVVH: Continuous venovenous haemofiltration
CVVHDF: Continuous venovenous haemodiafiltration
*f* T _> MIC_: Percentage of time remaining concentration above MIC
FTA: Fractional target attainment
HPLC-UV: High-performance liquid chromatography with UV-detection
ICU: Intensive care unit
IHD: Intermittent haemodialysis
IQR: Interquartile range
KCP: Constant for meropenem distribution from central to peripheral compartment
KPC: Constant for meropenem distribution from peripheral to central compartment
MIC: Minimal inhibitory concentration
N/A: Not applicable
NPAG: Nonparametric adaptive grid
NPDE: Normalised prediction distribution errors
PCT: Procalcitonin
PD: Pharmacodynamic
PI: Prolonged infusion
PK: Pharmacokinetic
PTA: Probability of target attainment
RRT: Renal replacement therapy
R2: Linear regression
RD: Residual diuresis
SD: Standard deviation
SLED: Sustained low-efficiency dialysis
SOFA: Modified sequential organ failure assessment score
SAPS II: Simplified acute physiology score
TVCL: Typical value of total clearance
V_c_: Volume of distribution of the central compartment
VPC: Visual predictive check
